# Relationship of Cerebrospinal Fluid Vitamin B12 Status Markers With Parkinson's Disease Progression

**DOI:** 10.1002/mds.28073

**Published:** 2020-05-14

**Authors:** Chadwick W. Christine, Peggy Auinger, Nasrin Saleh, Miao Tian, Teodoro Bottiglieri, Erland Arning, Nam K. Tran, Per Magne Ueland, Ralph Green, the Parkinson Study Group—DATATOP Investigators

**Affiliations:** ^1^ Department of Neurology UCSF San Francisco California USA; ^2^ Center for Health and Technology University of Rochester Rochester New York USA; ^3^ Department of Pathology and Laboratory Medicine UC Davis Sacramento California USA; ^4^ Institute of Metabolic Disease, Baylor Scott & White Research Institute Dallas Texas USA; ^5^ Bevital, New Lab Building 9th floor Bergen Hordaland Norway

**Keywords:** cognitive impairment, CSF, cyanocobalamin, gait instability, hyperhomocysteinemia, vitamin B12

## Abstract

**Background:**

Using blood specimens from untreated early Parkinson's disease (PD) patients from the DATATOP trial, we found that subjects in the low serum vitamin B12 tertile experienced greater annualized change in ambulatory capacity score, whereas those with moderately elevated (>15 μmol/L) total homocysteine had greater annualized declines in the Mini‐Mental State Exam.

**Methods:**

In this this study we sought to determine whether levels of cerebrospinal fluid (CSF) B12 markers were also associated with progression of PD.

**Results:**

The annualized change in the UPDRS “walking” item, a component of the ambulatory capacity score, was worse in the low B12 tertile. No association with change in the Mini‐Mental State Exam was seen for those 7% with the highest baseline CSF total homocysteine.

**Conclusions:**

In these untreated early‐PD subjects, low CSF B12 predicted greater worsening of the UPDRS “walking” item, whereas CSF total homocysteine was not associated with progression of cognitive impairment. These findings extend and partially support our findings in serum. © 2020 The Authors. *Movement Disorders* published by Wiley Periodicals, Inc. on behalf of International Parkinson and Movement Disorder Society.

In our recent investigation of vitamin B12 status in untreated early Parkinson's disease (PD), using 680 baseline blood samples from the Deprenyl and Tocopherol Antioxidative Therapy of Parkinsonism (DATATOP) cohort,^1^ we found that those subjects with B12 levels in the low tertile developed greater morbidity, as measured by annualized changes in the ambulatory capacity score compared with those in the middle and upper tertiles. The ambulatory capacity score is a validated measure of gait and balance function calculated as the sum of 5 Unified Parkinson's Disease Rating Scale (UPDRS) items. Increases in the score reflect declines in gait function.^2^ Of equal interest, we found that those with moderately elevated total homocysteine (tHcy) had greater annualized declines in the Mini‐Mental State Exam (MMSE).

Although it is possible that the association of low B12 with more rapid impairment of ambulatory capacity is mediated by its known role supporting central and peripheral nervous system myelination, recent studies have shown that B12 inhibits α‐synuclein fibrillogenesis^3^ and that B12 allosterically modulates leucine‐rich repeat kinase 2 (LRRK2),^4^ an enzyme implicated in PD pathogenesis, raising a disease specific mechanism of action.

Because cerebrospinal fluid (CSF) is often considered the preferred biofluid to assess central nervous system status,^5^, ^6^ we sought to determine whether baseline CSF levels of B12, tHcy, and 2 other markers of B12 status, compared with previously obtained serum measurements, were more strongly associated with measures of disease progression in the DATATOP study.

## Methods

DATATOP was a double‐blind, randomized trial that tested whether treatment with deprenyl (selegiline hydrochloride) and/or the antioxidant alpha‐tocopherol slowed PD progression. The study enrolled 800 participants between 1987 and 1988.^7^ Eligible subjects had early PD and were excluded if they had begun PD medications, had severe tremor, or had dementia. After the baseline visit, subjects were evaluated every 3 months up to 24 months. At each visit, subjects were assessed for disability sufficient to require levodopa therapy (the primary end point) as well as other outcomes including the UPDRS and the MMSE.^2^

CSF was collected at the baseline visit after overnight bed rest. At the time of collection, the CSF was rapidly frozen for storage at −70°C. We used samples obtained from the middle or end of the CSF collection, which were not stored with metabisulfite preservative.

CSF B12 was measured using a microbiological assay.^8^ Samples were diluted 1:4, and following an extraction step diluted a further 1:1.2 for an overall dilution of 1:4.8. Methylmalonic acid (MMA) was measured using liquid chromatography–tandem mass spectrometry (LC‐MS/MS).^9^ Holotranscobalamin (holoTC) was determined by monoclonal antibody capture assay.^10^

tHcy was determined by LC‐MS/MS using a modification of a previously described method.^11^ Separation and detection of tHcy were performed with a Nexera UPLC system (Shimadzu, Kyoto, Japan) interfaced with a 5500 QTRAP (Sciex, Framingham, MA). All data were collected and processed using Analyst software version 1.6.2 (Sciex, Framingham, MA).

The annualized change in PD‐related motor scores and MMSE were determined based on the change from baseline to the primary end point or the final visit if the primary end point was not reached as previously described.^1^ Adjusted mean annualized change in scores (including UPDRS, ambulatory capacity, and MMSE) by baseline serum and CSF B12, MMA, holoTC, and tHcy tertiles and threshold cutoffs were compared using linear models that adjusted for baseline value of the outcome, sex, age, and treatment group. Pearson correlation coefficients were used to assess the associations among the 4 CSF analytes and between the analogous serum and CSF analytes. Multiple comparisons were accounted for by applying the Bonferroni correction in which *P* < 0.004 was considered statistically significant.

## Results

**Figure 1:**
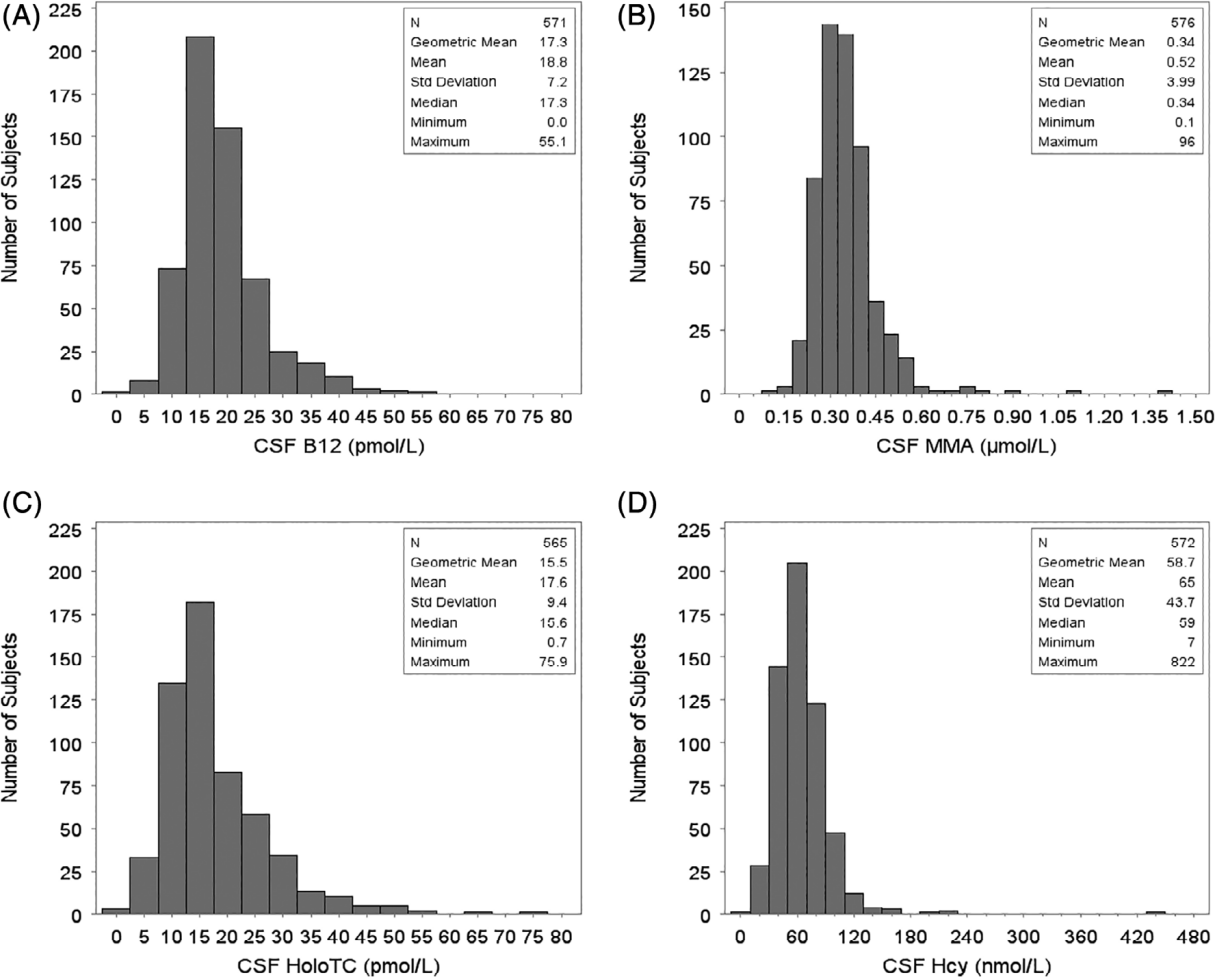
Distributions of CSF vitamin B12, MMA, HoloTc and tHcy.

Of 581 baseline CSF samples available, we measured B12 in 571 specimens, holoTC in 565 samples, MMA in 576, and tHcy in 572. The reason for the inability to measure analytes in samples from all participants was because of insufficient CSF volume. The geometric mean ± SD for CSF B12 was 17.3 ± 7.2 pmol/L, for CSF holoTC was 15.5 ± 9.4 pmol/L, for MMA was 0.34 ± 3.9 μmol/L, and for tHcy was 58.7 ± 43.7 nmol/L(Fig. 1).

CSF analytes showed significant associations with serum analytes, although the CSF levels were substantially lower than the serum levels for all except MMA. For example, CSF B12 compared with serum B12 measurements showed a correlation coefficient (*r*) of 0.57, *P* < 0.0001; however, the mean CSF B12 level was considerably lower, about 6% of the corresponding mean serum measurement (geometric mean of CSF B12/geometric mean of serum B12). CSF holoTC had an an *r* of 0.67 with serum holoTC, *P* < 0.0001, and mean CSF holoTC was 22% of mean serum levels. CSF MMA correlated with serum MMA with an *r* of 0.69, *P* < 0.0001, and mean CSF MMA was 170% of mean serum measurement. Finally, CSF tHcy had an *r* of 0.74 with serum tHcy, *P* < 0.0001; mean CSF tHcy was 0.6% of the mean serum measurement.

The most robust correlation between CSF B12 biomarkers was found between CSF B12 and CSF holoTC (eFig. 2), with an *r* of 0.87, *P* < 0.001. Weaker inverse correlations were found for CSF B12 and CSF MMA, *r* = −0.15, *P* = 0.0003, and CSF B12 and CSF tHcy, *r* = −0.18, *P* ≤ 0.0001.

### Associations of Baseline Serum and CSF Analytes With Clinical Progression

Table 1A shows that when the 570 serum samples, for which we had corresponding CSF samples, were analyzed according to baseline B12 tertiles, those subjects in the low B12 tertile developed significantly greater impairments (higher mean change scores) in ambulatory capacity compared with the middle and upper tertiles (1.61 compared with 0.91 and 0.67 points, respectively). Table 1B shows a similar relationship according to baseline CSF B12 measurements from these same subjects. In the CSF analysis, although the annualized change in the ambulatory capacity was greater in the low tertile compared with the high tertile, this difference was not significant. However, the annualized change in UPDRS “walking” score (1 of the 5 items that make up ambulatory capacity score) was significantly greater in the low tertile compared with the changes in either the high tertile or both middle and high tertiles combined (*P* < 0.004 for both comparisons).

**Table 1A.** Adjusted mean annualized change in outcomes according to tertiles of baseline **serum** B12 levels

	Baseline serum B12 tertile		
*Least‐squares mean annualized change outcome*	1^st^ (<233.2 pmol/L) n = 189	2^nd^ (233.2–321.7 pmol/L) n = 191	3^rd^ (>321.7 pmol/L) n = 190
UPDRS, total	14.37	11.71	10.72
UPDRS, part 1 (mental subscore)	0.81	0.44	0.53
UPDRS, part 2 (ADL subscore)	4.62	3.48	3.28
UPDRS, part 3 (motor subscore)	8.87	7.81	6.82
Ambulatory capacity	1.61^a,b^	0.91	0.67
Falling	0.18	0.09	0.002
Freezing when walking	0.18	0.11	0.06
Walking	0.44	0.29	0.21
Gait	0.37	0.32	0.30
Postural stability	0.41	0.17	0.14
MMSE	‐0.03	‐0.16	0.20

**Table 1B.** Adjusted mean annualized change in outcomes according to tertiles of baseline **CSF** B12 levels

	Baseline CSF B12 Tertile		
*Least‐squares mean annualized change outcome*	1^st^ (<15.31 pmol/L) (n = 190)	2^nd^ (15.31‐20.18 pmol/L) (n = 191)	3^rd^ (>20.18 pmol/L) (n = 190)
UPDRS, total	12.18	13.20	11.38
UPDRS, part 1 (mental subscore)	0.66	0.50	0.60
UPDRS, part 2 (ADL subscore)	4.26	4.00	3.13
UPDRS, part 3 (motor subscore)	7.38	8.46	7.62
Ambulatory capacity	1.22	1.25	0.72
Falling	0.15	0.09	0.02
Freezing when walking	0.11	0.20	0.04
Walking	0.45^a^^,^ b	0.32	0.16
Gait	0.30	0.33	0.36
Postural stability	0.24	0.31	0.17
MMSE	‐0.22	0.08	0.14

UPDRS Unified Parkinson's Disease Rating Scale; ADLs, activities of daily living; MMSE, Mini‐Mental State Exam.

dels are adjusted for baseline value of the outcome, age, sex, and treatment group.

a *P < 0.004 compared with third tertile.

b *P* < 0.004 compared with combined second and third tertiles.

Given the strong correlation between holoTC and B12 (eFig. 2), we performed an analysis of clinical progression according to serum and CSF holoTC. Trends for greater annualized worsening in the ambulatory capacity score according to serum and CSF levels of holoTC were observed but were not statistically significant (eTable 2). Although not statistically significant based on correction for multiple comparisons, the annualized change in the “walking” item was greater in both the low serum and low CSF holoTC tertiles, compared with the corresponding high tertile (*P* < 0.02; eTable 2A; *P* < 0.01; eTable 2B, respectively). No relationships for greater change in ambulatory capacity was found in the high serum or CSF MMA tertiles (data not shown).

Finally, we compared the change in MMSE scores according to thresholds of tHcy in serum and CSF. Although serum tHcy >15 μmol/L (39 subjects or 7%) predicted an annualized decline of 1.65 points in the MMSE compared with a 0.14‐point increase in those with tHcy ≤15 μmol/L, no association was observed in those subjects with the highest 7% of CSF tHcy (eTable 3).

## Discussion

In this secondary analysis of baseline serum and CSF analytes from a large cohort of early untreated PD patients, we found that serum and CSF analyte levels were directly associated and that the levels of B12, holoTC, and tHcy in CSF were much lower than in serum, whereas MMA was somewhat higher, as has been reported previously for normal subjects and patients with B12 deficiency.^12^ We also confirmed that in CSF, unlike serum, holoTC and B12 are strongly correlated, consistent with prior observations that in CSF, almost all B12 is bound to transcobalamin.^13^ Finally, those with low CSF B12 had greater declines in the UPDRS “walking” item (a component of the ambulatory capacity).

To our knowledge, this study is the largest study of serum and CSF analytes relating to B12 status in any PD cohort. CSF‐to‐serum analyte ratios in our study were 6% for B12, 22% for holoTC, 0.6% for tHcy, and 170% for MMA and are similar to the findings in prior studies.^14^, ^15^, ^16^ The substantially lower levels of B12 and holoTC in the CSF are consistent with the notion that B12 is supplied to the central nervous system by serum and not by way of the CSF.

Although we had hypothesized that CSF markers might be more sensitive predictors of PD progression because of the close anatomical relationship of CSF to the brain, our results do not support this hypothesis. This finding is similar to that observed regarding serum and CSF uric acid levels, with Ascherio and colleagues finding that serum uric acid measurements were a more sensitive predictor of PD progression compared with CSF levels.^17^ Because a recent study showed that blood‐CSF permeability is increased with PD progression,^18^ one explanation of why CSF levels of B12, holoTC, and tHcy are not as predictive as serum is that CSF levels of these analytes are raised because of increased blood‐CSF permeability. Although it remains possible that higher B12 levels are a nonspecific marker of better health status, we speculate that higher B12 levels may slow deterioration of gait either by reducing the development of neuropathy/myelopathy because of its known effect on nervous system myelination or possibly by affecting PD pathogenesis by reducing LRRK2 activity.^4^ Interestingly, prior studies have shown that B12 levels are lower in early PD^19^, ^20^ and decline more rapidly than expected in normal aging,^20^, ^21^ possibly because of reduced gastrointestinal absorption.^22^, ^23^

A potential limitation of this study was that the CSF was collected more than 30 years ago and that the analytes might not be stable over this time frame. However, because the DATATOP study specified strict procedures for CSF collection and storage and prior research demonstrating stability of B12,^24^ MMA,^24^ and tHcy^24^, ^25^ in frozen serum specimens for as long as 29 years, we have confidence that these CSF analyte measurements are valid.

These measurements in CSF support our prior work showing that lower serum B12 levels predict greater declines in ambulatory capacity in the DATATOP study.^1^ Further study of B12 and tHcy should be performed in contemporary PD cohorts and should also explore the relationship of B12 level with markers of LRRK2 kinase activity.^4^, ^26^

## Author Contributions

Chadwick W. Christine: research project ABC; statistical analysis AC; manuscript AB.

Peggy Auinger: research project BC; statistical analysis AB; manuscript B.

Nasrin Saleh: research project C; statistical analysis C; manuscript B.

Miao Tian: research project C; statistical analysis C; manuscript B.

Teodoro Bottiglieri: research project C; statistical analysis C; manuscript B.

Erland Arning: research project C; statistical analysis C; manuscript B.

Nam K Tran: research project C; statistical analysis C; manuscript B.

Per Magne Ueland: research project C; manuscript B.

Ralph Green: research project AB; statistical analysis AC: manuscript AB.

## Author Disclosures

(all sources of financial support for the preceding 12 months)

Dr. Christine reports research grants from Voyager Therapeutics, Inc, NINDS, and from Michael J. Fox Foundation.

Dr Auinger reports none.

Nasrin Saleh, Miao Tian, Teodoro Bottiglieri, Erland Arning, Nam K. Tran, and Per Magne Ueland: none.

Ralph Green reports a donation from an anonymous private foundation.

## Supporting information


**Table 2A** Adjusted Mean Annualized Change in Outcomes According to Tertiles of Baseline **Serum** Holo TC Levels
**Table 2B** Adjusted Mean Annualized Change in Outcomes According to Tertiles of Baseline **CSF** Holo TC Levels.

**Table 3A** Adjusted Mean Annualized Change in Outcomes According to Baseline **Serum** Total Homocysteine Levels
**Table 3B** Adjusted Mean Annualized Change in Outcomes According to Baseline **CSF** Total Homocysteine Levels

**Figure S2** Supporting information
