# Supplementary material for: Relationship of Cerebrospinal Fluid Vitamin B12 Status Markers With Parkinson's Disease Progression
Source: Mov Disord. 2020 May 14;35(8):1466–71. doi: 10.1002/mds.28073 (PMC7496300; doi:10.1002/mds.28073)
Supplement: Supplementary file 1 — Table 2A Adjusted Mean Annualized Change in Outcomes According to Tertiles of Baseline Serum Holo TC Levels Table 2B Adjusted Mean Annualized Change in Outcomes According to Tertiles of Baseline CSF Holo TC Levels. [file MDS-35-1466-s002.docx]

e-Table 2A

Adjusted Mean Annualized Change in Outcomes According to Tertiles of Baseline **Serum** Holo TC Levels

|  | Baseline Serum Holo TC Tertile | | |
| --- | --- | --- | --- |
| *Least Squares Mean Annualized Change Outcome* | 1^st^  (<58.5 pmol/L)  (n=190) | 2^nd^  (58.5-83.3 pmol/L)  (n=186) | 3^rd^  (>83.3 pmol/L)  (n=188) |
| UPDRS, total | 13.55 | 11.61 | 11.50 |
| UPDRS, Part 1 (Mental subscore) | 0.62 | 0.51 | 0.49 |
| UPDRS, Part 2 (ADL subscore) | 4.32 | 3.54 | 3.52 |
| UPDRS, Part 3 (Motor subscore) | 8.57 | 7.34 | 7.41 |
| Ambulatory capacity | 1.35 | 0.93 | 0.87 |
| Falling | 0.14 | 0.04 | 0.02 |
| Freezing when walking | 0.17 | 0.09 | 0.11 |
| Walking | 0.41* | 0.35 | 0.23 |
| Gait | 0.33 | 0.27 | 0.38 |
| Postural stability | 0.25 | 0.22 | 0.20 |
| MMSE | 0.10 | -0.40 | 0.11 |

*p-value=0.02 compared to 3^rd^ tertile

e-Table 2B

Adjusted Mean Annualized Change in Outcomes According to Tertiles of Baseline **CSF** Holo TC Levels.

|  | Baseline CSF Holo TC Tertile | | |
| --- | --- | --- | --- |
| *Least Squares Mean Annualized Change Outcome* | 1^st^  (<12.9 pmol/L)  (n=190) | 2^nd^  (12.9-18.6 pmol/L)  (n=186) | 3^rd^  (>18.6 pmol/L)  (n=189) |
| UPDRS, total | 12.72 | 13.23 | 11.04 |
| UPDRS, Part 1 (Mental subscore) | 0.61 | 0.53 | 0.60 |
| UPDRS, Part 2 (ADL subscore) | 4.10 | 4.17 | 3.15 |
| UPDRS, Part 3 (Motor subscore) | 8.08 | 8.32 | 7.25 |
| Ambulatory capacity | 1.33 | 1.11 | 0.78 |
| Falling | 0.15 | 0.07 | 0.04 |
| Freezing when walking | 0.16 | 0.18 | 0.01 |
| Walking | 0.41* | 0.35 | 0.18 |
| Gait | 0.35 | 0.29 | 0.37 |
| Postural stability | 0.26 | 0.26 | 0.20 |
| MMSE | -0.09 | 0.15 | -0.01 |

Models are adjusted for baseline value of the outcome, age, sex, and treatment group.

* p-value < 0.01 compared to 3^rd^ tertile.

UPDRS Unified Parkinson’s Disease Rating Scale; ADL, Activities of Daily Living; MMSE, Mini-Mental Status Exam.
