# Supplementary material for: Relationship of Cerebrospinal Fluid Vitamin B12 Status Markers With Parkinson's Disease Progression
Source: Mov Disord. 2020 May 14;35(8):1466–71. doi: 10.1002/mds.28073 (PMC7496300; doi:10.1002/mds.28073)
Supplement: Supplementary file 2 — Table 3A Adjusted Mean Annualized Change in Outcomes According to Baseline Serum Total Homocysteine Levels Table 3B Adjusted Mean Annualized Change in Outcomes According to Baseline CSF Total Homocysteine Levels [file MDS-35-1466-s003.docx]

**e-Table 3A** Adjusted Mean Annualized Change in Outcomes According to Baseline **Serum** Total Homocysteine Levels

|  | Baseline Serum Homocysteine | |  |
| --- | --- | --- | --- |
| *Least Squares Mean Annualized Change Outcome* | >15 µmol/L  n=39 | <15 µmol/L  n=530 | p-value |
| Total UPDRS | 10.98 | 12.48 | 0.63 |
| UPDRS, Part 1 (Mental subscore) | 0.72 | 0.57 | 0.70 |
| UPDRS, Part 2 (ADL subscore) | 4.12 | 3.81 | 0.77 |
| UPDRS, Part 3 (Motor subscore) | 6.28 | 8.03 | 0.40 |
| Ambulatory capacity | 1.03 | 1.09 | 0.91 |
| Falling | 0.09 | 0.09 | 0.98 |
| Freezing when walking | 0.29 | 0.10 | 0.12 |
| Walking | 0.33 | 0.31 | 0.88 |
| Gait | 0.24 | 0.35 | 0.46 |
| Postural stability | 0.19 | 0.25 | 0.74 |
| MMSE | -1.65 | 0.14 | 0.01 |

**e-Table 3B** Adjusted Mean Annualized Change in Outcomes According to Baseline **CSF** Total Homocysteine Levels

|  | Baseline CSF Homocysteine | |  |
| --- | --- | --- | --- |
| *Least Squares Mean Annualized Change Outcome* | >98 µmol/L  n=40 | <98 µmol/L  n=532 | p-value |
| Total UPDRS | 11.96 | 12.38 | 0.89 |
| UPDRS, Part 1 (Mental subscore) | 0.88 | 0.56 | 0.38 |
| UPDRS, Part 2 (ADL subscore) | 4.33 | 3.79 | 0.60 |
| UPDRS, Part 3 (Motor subscore) | 6.78 | 7.98 | 0.55 |
| Ambulatory capacity | 1.20 | 1.07 | 0.77 |
| Falling | 0.02 | 0.09 | 0.53 |
| Freezing when walking | 0.21 | 0.11 | 0.36 |
| Walking | 0.30 | 0.31 | 0.90 |
| Gait | 0.45 | 0.34 | 0.41 |
| Postural stability | 0.25 | 0.24 | 0.96 |
| MMSE | -0.39 | 0.06 | 0.50 |

Models are adjusted for baseline value of the outcome, sex, baseline age, and treatment group.

UPDRS Unified Parkinson’s Disease Rating Scale; ADL, Activities of Daily Living; MMSE, Mini-Mental Status Exam.
