# Supplementary figures and images for: Relationship of Cerebrospinal Fluid Vitamin B12 Status Markers With Parkinson's Disease Progression
Source: Mov Disord. 2020 May 14;35(8):1466–71. doi: 10.1002/mds.28073 (PMC7496300; doi:10.1002/mds.28073)

**Figure 2**: Relationship of CSF vitamin B12 with CSF holotranscobalamin


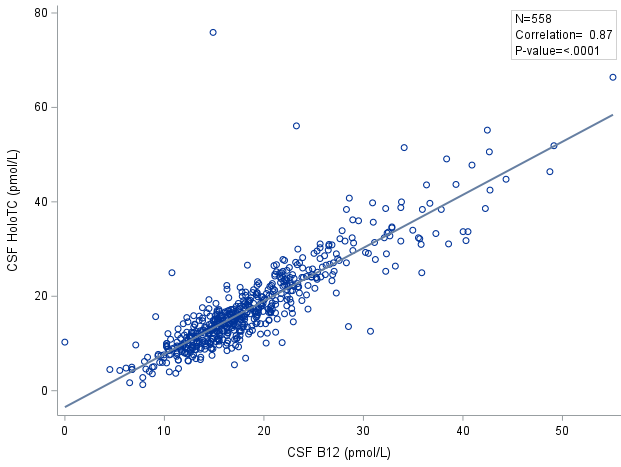

Supplement: Supplementary file 3 — Figure S2 Supporting information [file MDS-35-1466-s001.docx]
